# Use of the GlideScope®-Ranger for pre-hospital intubations by anaesthesia trained emergency physicians – an observational study

**DOI:** 10.1186/s12873-016-0069-2

**Published:** 2016-01-29

**Authors:** Sebastian G. Russo, Eike A. Nickel, Kay B. Leissner, Katrin Schwerdtfeger, Martin Bauer, Markus S. Roessler

**Affiliations:** Department of Anaesthesiology, University Hospital Göttingen, 370799 Göttingen, Germany; Current address: Department of Anaesthesiology and Pain Medicine, HELIOS Klinikum Emil-von-Behring, Berlin, Germany; Department of Anesthesiology, VA Boston Healthcare System, Harvard Medical School, Boston, MA USA

**Keywords:** Airway management, Pre-hospital, Videolaryngoscopy, GlideScope, Intubating laryngeal mask airway

## Abstract

**Background:**

Pre-hospital endotracheal intubation is more difficult than in the operating room (OR). Therefore, enhanced airway management devices such as video laryngoscopes may be helpful to improve the success rate of pre-hospital intubation. We describe the use of the Glidescope®-Ranger (GS-R) as an alternative airway tool used at the discretion of the emergency physician (EP) in charge.

**Methods:**

During a 3.5 year period, the GS-R was available to be used either as the primary or backup tool for pre-hospital intubation by anaesthesia trained EP with limited expertise using angulated videolaryngoscopes.

**Results:**

During this period 672 patients needed pre-hospital intubation of which the GS-R was used in 56 cases. The overall GS-R success rate was 66 % (range of 34–100 % among EP). The reasons for difficulties or failure included inexperience of the EP with the GS-R, impaired view due to secretion, vomitus, blood or the inability to see the screen in very bright environment due to sunlight.

**Conclusion:**

Special expertise and substantial training is needed to successfully accomplish tracheal intubation with the GS-R in the pre-hospital setting. Providers inexperienced with DL as well as video-assisted intubation should not expect to be able to perform tracheal intubation easily just because a videolaryngoscope is available. Additionally, indirect laryngoscopy might be difficult or even impossible to achieve in the pre-hospital setting due to impeding circumstances such as blood, secretions or bright sun-light. Therefore, videolaryngoscopes, here the GS-R, should not be considered as the “Holy Grail” of endotracheal intubation, neither for the experts nor for inexperienced providers.

**Electronic supplementary material:**

The online version of this article (doi:10.1186/s12873-016-0069-2) contains supplementary material, which is available to authorized users.

## Background

Oxygenation and ventilation of the patient’s lungs are crucial for survival of critically ill patients in the pre-hospital setting. Several authors have emphasized the challenges as well as the alarmingly high failure rates for pre-hospital endotracheal tube (ETT) placement [1–4]. In contrast, other authors have described more favourable success rates when pre-hospital airway management was performed by anaesthesiologists or skilled emergency physicians [5–7]. Providers less skilled in endotracheal intubation have therefore been encouraged to use supraglottic airway devices for oxygenation and ventilation and to abandon ETT placement [8]. Nevertheless, endotracheal intubation is considered the gold standard of airway management, because it most reliably secures and protects the airway, allows higher ventilation pressures and has proved to be associated with better outcome in cardiac arrest [9]. Direct laryngoscopy (DL) has been the most commonly used technique for pre-hospital tracheal intubation.

Numerous studies have shown, that the GlideScope® videolaryngoscope (GS-VL, Verathon Inc., Bothell, WA, USA), provides significant better exposure of the laryngeal structures in normal and difficult to manage airways compared to DL, resulting in an improved glottic exposure during ETT placement [10–12]. A manikin study suggested that a low number of approximately six successfully performed tracheal intubations may be adequate to gain proficiency [13]. Furthermore, one study has demonstrated that acquiring intubation skills by untrained medical personal was enhanced by GS-VL use [14].

As discussed extensively in the literature, the conditions for ETT placement in the pre-hospital setting are different compared to the conditions in emergency departments (ED) or in the operating room (OR) [15]. Location and position of patients are often suboptimal for DL outside a hospital and secretions or fluids such as blood or vomitus may impair visualization of the glottic inlet. So far, a limited number of studies is available about the use of videolaryngoscopes in the pre-hospital setting [16, 17]. Cavus et al. evaluated the C-Mac videolaryngoscope (Karl Storz, Tuttlingen, Tuttlingen, Germany) in a non-hospital setting and were unable to perform videolaryngoscopic assisted endotracheal intubation in 7.5 % of patients, primarily because of obstructed glottic views due to secretions and blood [18].

In this study, we evaluated the use of the GlideScope®-Ranger (GS-R, Verathon Inc., Bothell, WA, USA), a portable version of the GS-VL during consecutive pre-hospital intubations performed by physician anaesthesiologists, who were experienced in emergency and advanced airway management, but had individually differing levels of expertise with the GS-VL.

## Methods

After approval of the local ethical committee of the University of Göttingen, Medical School (approval number 13/3/09), we prospectively evaluated pre-hospital emergency intubations performed by anaesthesia trained emergency physicians (EP) from July 2009 until December 2012. As the indication to perform tracheal intubation was dictated by the patients’ medical conditions within the emergency setting the ethical committee waived the need for patient consent during this anonymous evaluation.

**Fig. 1:**
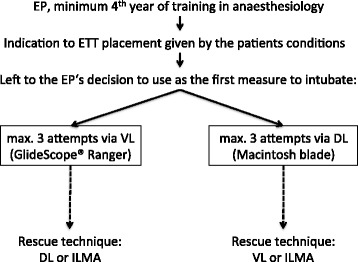
Flow-chart of the study., EP = Emergecny Physician, ETT = endotracheal tube, VL = videolaryngoscopy, DL = direct laryngoscopy, ILMA = intubating laryngeal mask airway

All EP were at least in their fourth year of anaesthesia training and had performed a minimum of five successful videolaryngoscope assisted tracheal intubations with the GS-R in the OR. The majority of EP had an experience of less than twenty intubations using a videolaryngoscope. We recorded all GS-R intubation attempts by the EP while they were on duty either on emergency medical services (EMS) ambulances or on EMS helicopters. The indication for endotracheal intubation was at the discretion of the EP based on the patient’s clinical condition. Since the GS-R was not considered to be used exclusively as an escalation strategy in difficult airway management, the EP were free to use either DL with a standard single-use Macintosh blade (size 3) or videolaryngoscopy with the GS-R (size 3 blade) as the primary tool for ETT placement in order to ensure prompt and high quality emergency endotracheal intubation. Additional blades in different sizes were available for initial intubation attempts, or for subsequent intubation attempts with DL or GS-R guided intubation when indicated. Because of varying levels of experience with the videolaryngoscope and heterogeneous types of emergencies the patients were not randomized. For GS-R guided intubation the GlideRite (semi-) rigid stylet (Verathon Medical, Verathon Inc., Bothell, WA, USA) was used in all cases. The study protocol is described in Fig. 1.

Successful and correct ETT placement was confirmed by capnography, visualization of the tube passing through the vocal cords, as well a by auscultation.

Moreover, we recorded the type of emergency, estimated patient’s body weight and height, the presence of upper or lower dentures, signs of an expected difficult airway (DA) and whether induction of general anaesthesia was performed prior to intubation. Our primary outcome parameters were the Cormack and Lehane grade (C&L), number of attempts and success rates of tracheal intubation. Secondary outcome parameters were the level of difficulty for blade insertion, laryngoscopy and intubation (rated as easy, moderate, difficult or impossible). Furthermore, the EP had to rate the ease of operating the laryngoscope (DL or GS-R) on a numeric analogue scale from 1 (very easy) to 10 (very difficult).

If laryngoscopy or intubation proofed to be difficult, the EP had to indicate and describe the experienced problems in a free-text format. These problems were categorized as: 1) failure during laryngoscopy (e.g. due to secretion, blood or due to technical problems such as low battery or inability to visualize any laryngeal structures due to reflection of bright sun light), 2) failure to intubate despite visualisation of the glottis or 3) distinct patient characteristics which would have made ETT placement difficult during either direct or video-assisted laryngoscopy (e.g. swelling of the laryngeal structures). Failure to successfully place an ETT despite video-assisted visualisation of the glottis was considered failure to intubate due to insufficient proficiency of the EP with the technique of indirect laryngoscopy.

Induction of anaesthesia was left to the EP discretion depending on the clinical situation. The standard medication available on the ambulance for anaesthesia induction are fentanyl as analgesic, propofol, etomidate or midazolam as anaesthetics/sedatives and suxamethonium or rocuronium as neuromuscular blocking agents.

The manuscript respects the STROBE (Strengthening the reporting of observational studies in epidemiology) checklist, which is available as an Additional file 1 of the publication.

Statistics: Data are presented as mean ± SD and have been analysed descriptively using Excel for Mac 2011.

## Results

During the study period of 42 months 33 different EP were staffing the EMS and could have used the GS-R for pre-hospital airway management. Thirteen EP used the GS-R at least once (minimum once, maximum 11 uses; Table 1), whereas twenty EP did not attempt to use the GS-R due to lack of comfort using the device. During the study period 672 intubations occurred. Of these 56 (8 %) were attempted primary via GS-R without initial DL attempt.

**Table 1:** Numbers of attempts, failures and reasons to fail for each emergency physician

			Reasons for a failed GlideScope® Ranger aided intubation		
EP	Patients attempted to intubate with GS-R	Failures	Laryngoscopy	Proficiency	Patient
1	2	1	1^L^		
2	8	3	2^S^ + 1^L^		
3	9	2	2^S^		
4	11	0			
5	4	3	2^S^	1	
6	2	0			
7	1	0			
8	2	0			
9	3	2	2^S^		
10	1	0			
11	3	2	1^S^	1	
12	9	5	2^S^	2	1
13	1	1		1	

^S^failure due to regurgitation/secretion; ^L^ failure due to sunlight

If ETT placement via direct laryngoscopy (DL-ETT) failed after a maximum of three attempts, blind intubation via the intubating laryngeal mask airway (ILMA, Teleflex Inc., LMA Deutschland, Bonn, Germany) was used as the primary rescue technique for tracheal intubation. The GS-R was not used as a rescue device in case of a failed tracheal tube placement primarily attempted via DL.

### Videolaryngoscopy as the first measure

The mean age of patients, in which GS-R use was attempted initially for ETT placement, was 63 ± 20 years with an estimated height and weight of 174 ± 8 cm and 82 ± 18 kg, respectively. The main clinical indication to use the GS-R was cardiac arrest (*n* = 22 of 56 cases (39 %)) followed by patients with multiple injuries (polytrauma) requiring advanced airway management (*n* = 11; 20 %), other (*n* = 10; 18 %), neurological disorders (*n* = 8; 14 %) and isolated traumatic brain injury (TBI; *n* = 5; 9 %).

Out of 56 GS-R aided tracheal intubations ultimately 37 were successful (66 %). Of these, four (*n* = 4 of 37 (11 %)) were rated as difficult, 13 (35 %) as moderate difficult and 20 (54 %) as easy based on the patient’s airway anatomy.

**Fig. 2:**
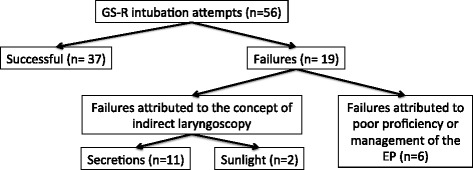
Presentation of the 56 GlideScope® Ranger aided intubation attempts. GS-R = GlideScope® Ranger

Nineteen GS-R aided intubation attempts failed (*n* = 19 of 56 (34 %)). In eleven of these cases (*n* = 11 of 19 (58 %)) secretions inhibited the view of the laryngeal structures. Excessive sunlight blinded the view of the video-monitor in two cases (10.5 %). ETT placement failed in five cases (5 of 19 (26 %)) despite successful visualization of the glottic opening with a C&L grade equal or better than 2, which was considered to be due to inadequate proficiency in GS-R use by the EP (Table 1). In addition, insufficient depth of anaesthesia was reported in one patient, resulting in failed GS-R intubation. After administration of neuromuscular block agents (NMB), ETT placement was successfully achieved with DL in this patient. Excluding this latter patient as well as those cases, in which inadequate proficiency of the provider might have been a contributing factor, 13 of 56 (23 %) GS-R intubation attempts failed, because of problems that are more likely to appear with indirect laryngoscopy (see Fig. 2).

If visualization was not impaired by sunlight or airway secretions, the observed videolaryngoscopic C&L grades were distributed as follows: C&L I 70 % (30 out of 43), C&L II 19 % (8 out of 43), C&L III 9 % (4 out of 43) and C&L IV 2 % (1 out of 43).

In 23 patients (*n* = 23 of 56 (41 %)) the EP determined the airway to be potentially difficult to manage; either because of the patient position on the floor, micrognathia, short thyro-mental distance, short neck or cervical spine immobilisation.

After a failed GS-R intubation attempt the use of the ILMA succeeded in six and DL in 13 cases. For a detailed presentation of the failed intubation attempts see Table 2.

**Table 2:** Characteristic of the failed intubation during indirect laryngoscopy with the GlideScope® Ranger (Verathon Medical, Verathon Inc., Bothell, WA, USA)

Patient	Indication	DA expected prior to attempt ETT	Induction of anaesthesia	NMB	Number of laryngoscopy attempts with the GS-R	best C&L-Grade via GS-R	Intubation failed due to…	Rescue technique	C&L-Grade via DL
1	CPR	No	No	No	2	II	Proficiency	DL	II
2	Neuro	Yes^a^	Yes	Yes	1	n.a.	Regurgitation / Secretion	ILMA	n.a.
3	CPR	No	No	No	3	n.a.	Regurgitation / Secretion	DL	I
4	PT	No	No	No	2	n.a.	Sun light	ILMA	n.a.
5	PT	No	Yes	Yes	2	n.a.	Regurgitation / Secretion	ILMA	n.a.
6	Others	Yes^b^	No	No	3	n.a.	Regurgitation / Secretion	DL	II
7	CPR	No	No	No	1	n.a.	Regurgitation / Secretion	DL	II
8	CPR	Yes^c^	No	No	1	n.a.	Regurgitation / Secretion	DL	II
9	TBI	No	Yes	Yes	1	I	Proficiency	ILMA	n.a.
10	CPR	No	No	No	1	n.a.	Regurgitation / Secretion	DL	II
11	CPR	Yes^d^	No	No	1	II	Proficiency	ILMA	n.a.
12	TBI	No	Yes	Yes	1	n.a.	Sun light	DL	I
13	PT	Yes^e^	Yes	Yes	1	n.a.	Regurgitation / Secretion	DL	I
14	PT	Yes	Yes	No	2	n.a.	Lack of NMB	DL (+NMB)	I
15	PT	Yes^f^	Yes	Yes	2	I	Proficiency	DL	II
16	CPR	Yes^g^	No	No	2	n.a.	Regurgitation / Secretion	DL	I
17	CRP	No	No	No	1	n.a.	Regurgitation / Secretion	DL	II
18	Other	Yes^h^	Yes	Yes	1	III*	Regurgitation / Secretion	ILMA	n.a.
19	CPR	No	No	No	1	II	Proficiency	DL	m.v.

*DA* difficult airway, *ETT* endotracheal tube, *PT* polytrauma, *TBI* traumatic brain injury, *CRP* cardiopulmonary resuscitation, *Neuro* neurological disorders (e.g. ischemic, intracerebral bleeding), *NMB* neuromuscular block; ^a^Position of the patient; ^b^micrognathia and obesity; ^c^known massive regurgitation prior to attempt intubation, ^d^micrognathia; ^e^cervical spine immobilisation and blood in the oral cavity; ^f^short neck, blood in the oral cavity; ^g^short neck and obesity; ^h^no cervical spine movement due to anchylosing spondylitis; *best view achieved prior to regurgitation

The overall level of difficulty for blade insertion was rated as easy in 41 out of 56 (73 %) cases and moderate in 15 out of 56 (27 %) patients. Laryngoscopy was rated as follows: easy *n* = 29 (52 %), moderate *n* = 8 (14 %), difficult *n* = 6 (11 %), impossible *n* = 13 (23 %). The mean ease of operating the laryngoscope was 3.1 (min 1, max 10, median 2) on the numeric analogue scale.

Selected comments extracted out of the EP’s free-text comment section are presented in Table 3 to highlight potential pitfalls and strengths of the GS-R in the pre-hospital setting.

**Table 3:** Selected written comments of the EPs as examples to highlight the pros and cons of indirect videolaryngoscopy

Example	Fee-text comment
1	Secretion obstructed view despite repetitive suction attempts
2	Insertion of GS-blade size #3 → view obstructed due to secretion → DL → removal of secretion by direct suction → C&L III during DL → re-insertion of GS-blade size #3 → blade to small → insertion of GS-blade size #4 → C&L IIa → tracheal intubation
3	Secretion with the GS-R in place obscures view → Equipment: flexible suction catheter → small mouth opening in combination with GS-blade inserted in the oral cavity makes the insertion and proper placement of suction catheter impossible
4	Very comfortable to intubate with the patient on the floor during CPR during continuous external chest compression. No direct optical axis necessary.
5	Bright sunlight makes view on the monitor impossible
6	Car accident, EP placed behind the driver on the back seat. Driver with life-threatening airway (A) and breathing (B) problem. Paramedic holding the monitor of the GS-R, intubation performed by the EP while sitting behind the driver on the back seat. Difficult but possible, would not have been possible using DL.

## Discussion

We describe the use of the GS-R in the pre-hospital setting by anaesthesia trained EP with broad experiences for DL, but varying experience with the GL-VL. Our main findings include: 1) Despite encouraging results about steep learning curves and how easy intubation by using videolaryngoscopes can be, especially for novice users [13, 14], we have shown in this study that it required detailed knowledge and practice with the device to achieve reliable success rates for tracheal intubation under more difficult conditions, e.g. outside the hospital. This includes EP with broad experience in DL-guided intubation. 2) Common clinical scenarios of emergency patients in the pre-hospital setting – such as secretions or blood in the oropharynx – might impede the use of videolaryngocopes.

Our results emphasize that the pre-hospital conditions for airway management with the GS-VL are dissimilar compared to the OR setting [15, 19]. Even though all EP in this study were anaesthesiologists our data suggest that a minimum of five successful in-hospital intubations are insufficient to gain appropriate skills using the GS-VL for advanced airway management in challenging circumstances. Moreover, our results contradict data from a manikin study suggesting that approximately six intubations with an angulated VL might be enough to gain VL proficiency [13].

There are only a limited number of published studies evaluating the GS-VL in the pre-hospital setting [16, 17]. Nouruzi-Sedeh et al. report success rates of 95–100 % by novice users in the OR after two practice attempts of all GS-VL intubations without any failed intubations [14]. Struck and colleagues evaluated the GS-VL in an EP-based emergency medical system, which was similar compared to our study [17]. The authors describe that 15 % of all intubations during their study period were performed using the GS-VL instead of DL. In contrast to our study, all GS-VL intubations were ultimately successful. In their study Struck et al. reported difficulties mainly during intubation of trauma patients, especially with cervical spine immobilisation. In our study a major concern was regurgitation and secretion impairing the view on the laryngeal inlet, most often during cardiopulmonary resuscitation (CPR).

Most of the pre-hospital intubations in our evaluation were preformed using DL, despite the availability of the GS-R, most likely because the EP felt less comfortable using the GS-VL in an emergency. This is also supported by the fact that 20 out of 33 EP did not use the GS-R at all, but relayed on the traditional DL. Indeed, even if DL-ETT failed, the GS-R was not the primary back-up device.

The ILMA had been implemented in our EP practice for many years. Our study as well as previous reports have demonstrated the usefulness of the ILMA in cases of difficult or failed intubations [1, 20]. A number of the EPs preferred to use the ILMA as a rescue device since ventilation of the patient is typically achieved directly after device insertion prior to ETT placement. In our study this was the case even in a patient suffering from ankylosing spondylitis with severe deformity, in whom the best achievable glottis view was C&L Grade 3 using the GS-VL (see Patient No 18). During the study period the ILMA has been used a total of 14 times. Six of the intubations using the ILMA have been performed after a failed GS-R intubation attempt (six out of 56), eight out of 616 times after failed DL.

Without any doubt, proficiency, experience and training play a crucial role not only in primary selection of the laryngoscopy instrument, but also in first pass and overall success rates. This is demonstrated by the fact that EP #4 (SGR), who had performed more than 50 successful video-assisted tracheal intubations prior to start the study, used the GS-R as the primary device in all pre-hospital intubations with a 100 % success rate (see also Table 1). However, despite adequate proficiency certain limitations of indirect laryngoscopy may lead to failure of tracheal intubation using a VL. In an out-of-hospital situation bright sunlight may impair the view of the video screen. Even more important, secretions or fluids on the lens or optical chip may render the video-assisted view inadequate for successful intubation. Indeed, the latter was the main reason for most failed GS-R intubations in our study. The obstacles we found in our study match the difficulties Cavus et al. reported for the pre-hospital use of the CMac-VL [18] as well as for those described by Helm et al. for DL [21]. A helpful and important standard might be to thoroughly suction the oropharynx prior to the insertion of a VL, which was not a common practice during our evaluation. Our results, therefore, need to be interpreted with caution, as we cannot exclude that the view on the larynx might have been better for some patients. As no straight optical axis will be generated during GS-VL use, it is essential to have a rigid and kinked suction device available (e.g. a Yankauer suction tip) in order to suction in front of the glottic inlet. Training of EMS personnel therefore must include airway suction techniques. Furthermore, appropriate suction devices need to be readily available in the pre-hospital setting.

Since extreme situation, e.g. heavy bleeding into the oral cavity, will make the use of a VL impossible alternative techniques are still needed for tracheal intubation and may be life saving in some situations. Whether devices, which allow both a direct laryngoscopic and videolaryngoscopic view, for example videolaryngoscopes with a Macintosh-type blade [18] prove to be the favourable videolaryngoscopic technique in the pre-hospital setting is not yet know.

Nevertheless, given the challenging conditions in the pre-hospital setting (limited access to the patient’s head, unfavourable position of the patient) the GS-R was the superior option for some patients; for example, ETT placement in an entrapped patient in the back seat after a car accident (Table 3, example 6).

As the main limitation of our study we need to acknowledge that we are presenting ‘self reported data’. This may lead to potentially biased reports, e.g. the rate of successful intubations may be falsely high since this also addresses the performance of the EP. Furthermore, there might be a bias to disfavour the GS-R as the EP had only a limited experiences using the GS-VL and felt very comfortable with DL as well as using the ILMA.

## Conclusion

The GS-VL can be a helpful device for pre-hospital airway management, especially if laryngoscopy proves to be very difficult. Indirect laryngoscopy, however might be difficult or even impossible in the pre-hospital setting due to impeding circumstances such as blood, secretions or bright sun-light. Based on our results, providers inexperienced with DL as well as video-assisted intubation should not expect to be able to perform tracheal intubation in the field just because a videolaryngoscope is available. Thorough experience with video-assisted intubation in non-emergencies is essential prior to its use during emergencies. Therefore, VLs such as the GS-VL, should not be considered as the “Holy Grail” of endotracheal intubation, neither for the experts nor for inexperienced providers [22]. In the era of videolaryngoscopy techniques such as DL, use of the ILMA and other supraglottic airway devices remain valuable first line or rescue strategies for emergency airway management.

## Additional file

Additional file 1:
**STROBE Statement—checklist of items that should be included in reports of observational studies.** (PDF 94 kb)

## Abbreviations

C&L: cormack & lehane
DA: difficult airway
DL: direct laryngoscopy
DL-ETT: ETT placement via direct laryngoscopy
EMS: emergency medical system
EP: emergency physician
ETT: endotracheal tube
GS-R: GlideScope® Ranger
GS-VL: GlideoScope® videolaryngoscope
ILMA: intubating laryngeal mask airway
NMB: neuromuscular block
